# Interactions with fungi vary among *Tripsacum dactyloides* genotypes from across a precipitation gradient

**DOI:** 10.1093/aobpla/plad072

**Published:** 2023-11-02

**Authors:** Ceyda Kural-Rendon, Natalie E Ford, Maggie R Wagner

**Affiliations:** Department of Ecology and Evolutionary Biology, University of Kansas, Lawrence, KS 66045, USA; Department of Ecology and Evolutionary Biology, University of Kansas, Lawrence, KS 66045, USA; Kansas Biological Survey and Center for Ecological Research, University of Kansas, Lawrence, KS 66045, USA; Department of Ecology and Evolutionary Biology, University of Kansas, Lawrence, KS 66045, USA

**Keywords:** Endophytes, fungi, local adaptation, perennial, plant microbiome, precipitation, stress gradient hypothesis, tallgrass prairie, *Tripsacum dactyloides*

## Abstract

Plant-associated microbes, specifically fungal endophytes, augment the ability of many grasses to adapt to extreme environmental conditions. *Tripsacum dactyloides* (Eastern gamagrass) is a perennial, drought-tolerant grass native to the tallgrass prairies of the central USA. The extent to which the microbiome of *T. dactyloides* contributes to its drought tolerance is unknown. Ninety-seven genotypes of *T. dactyloides* were collected from native populations across an east–west precipitation gradient in Kansas, Oklahoma and Texas, and then grown together in a common garden for over 20 years. Root and leaf samples were visually examined for fungal density. Because fungal endophytes confer drought-tolerant capabilities to their host plants, we expected to find higher densities of fungal endophytes in plants from western, drier regions, compared to plants from eastern, wetter regions. Results confirmed a negative correlation between endophyte densities in roots and precipitation at the genotype’s original location (*r* = −0.21 *P* = 0.04). Our analyses reveal that the host genotype’s origin along the precipitation gradient predicts the absolute abundance of symbionts in the root, but not the relative abundances of particular organisms or the overall community composition. Overall, these results demonstrate that genetic variation for plant–microbe interactions can reflect historical environment, and reinforce the importance of considering plant genotype in conservation and restoration work in tallgrass prairie ecosystems.

## Teaser

Individuals of *Tripsacum dactyloides* (Eastern Gamagrass), a tallgrass prairie native, collected across the central USA retain distinct genotypic and local adaptation patterns long after transplanting into a single garden with a common environment. We found individuals of *T. dactyloides* originally from drier areas had higher densities of fungal endophytes in their roots. This demonstrates that plants are able to selectively recruit microbes from their immediate environment in a way that reflects their historic environment due to the plant’s genetics. This information can be crucial to consider for tallgrass prairie conservation and restoration, where plants can be sourced from locations with similar climates to increase resiliency.

## Introduction

As climates warm globally, the productivity and functionality of both natural and agricultural ecosystems will be significantly altered (Emadodin *et al.* 2021; White *et al.* 2021). Coinciding with warming trends, the frequency and severity of drought have increased and are expected to worsen (Trenberth 2011; Balting *et al.* 2021; Chiang *et al.* 2021). Although changes in precipitation can affect all ecosystems, a study conducted by Cherwin and Knapp (2012) in New Mexico and Colorado determined that semi-arid grasslands, such as those found in the Great Plains region of the USA, may be more prone to periods of drought than others. Within the Great Plains region, tallgrass prairie ecosystems contain many drought-tolerant grass species (Tucker *et al.* 2011). Native tallgrass prairies of the US Great Plains were once the dominant ecosystem (Bock and Bock 1998), but anthropogenic encroachment has resulted in a drastic reduction in land area, such that only about 1 % of the original tallgrass prairie remains (Cully *et al.* 2003). As periods of drought become more recurrent, plants in both natural and agricultural systems will need to adapt to survive. Understanding the drought tolerance of the perennial grasses that dominate the Great Plains region can have important implications for other drought-sensitive grasses and annual crops.

The ability of a population of plants to locally adapt is imperative to its survival under stressful changing climatic conditions (Lortie and Hierro 2022). When a genotype native to an area fares better in its native environment compared to genotypes that are not native to the same area, the native genotypes are said to be locally adapted (Clausen *et al.* 1941; Leimu and Fischer 2008). Over time, especially in stressful environments, natural selection increases the frequency of alleles that enhance survival and reproduction in a given environment.

In addition to local adaptation to the abiotic environment, interactions with microbial neighbours also affect plants’ ability to mitigate stress. It is well-documented that plants interact with a microbiome that spans and is differentiated between compartments such as the rhizosphere, phyllosphere and endosphere (Vorholt 2012; Hardoim *et al.* 2015; Compant *et al.* 2019). These microbial communities, comprised of both endophytes and epiphytes, are important for plant growth and survival through microbe-mediated pathogen defense and nutrient uptake (Bulgarelli *et al.* 2013; Vandenkoornhuyse *et al.* 2015). For example, microorganisms in different plant compartments can confer tolerance to abiotic stresses like drought (Poudel *et al.* 2021), extreme temperatures (Ali *et al.* 2009), salinity (Barassi *et al.* 2006) and metal toxicity (Dary *et al.* 2010). Furthermore, research suggests that most, if not all, plants harbour fungal endophytes in nearly all compartments (Weiß *et al.* 2011; Khare *et al.* 2018; Grabka *et al.* 2022), and these endophytes can have significant effects on host fitness (Rodriguez *et al.* 2009). Fungal endophytes can help their hosts adapt to environmental stressors such as a period of drought, specifically by strengthening the host’s matrix potential and/or reducing the host’s sensitivity to harmful reactive oxygen species (Rodriguez *et al.* 2008). Distinct from fungal endophytes, which live entirely within the plant (Rodriguez *et al.* 2009), are arbuscular mycorrhizal fungi (AMF), which partially reside within the roots of the host plant, but have hyphae that extend into the soil that can improve plant nutrient and water uptake and confer resistance to both biotic and abiotic stressors (Begum *et al.* 2019). Similarly, the dependence of the prairie grass *Andropogon gerardii* (Big bluestem) on AMF depends on the availability of nutrients in the soil, suggesting that AMF-infected roots are more effective at resource acquisition compared to the plant roots alone (Schultz *et al.* 2001).

The processes of local adaptation and host–microbiome interactions are not independent. Recent research has shown evidence for the heritability of plant–microbe associations (Peiffer *et al.* 2013; Walters *et al.* 2018; Deng *et al.* 2021), indicating that genetically variable plant traits promote associations with certain microorganisms, which may, in turn, affect host fitness in the local environment. Importantly, fungal endophytes can reinforce local adaptation of their host. For instance, Gibert *et al.* (2012) found higher infection rates of beneficial fungal endophytes in populations of *Lolium perenne* (perennial ryegrass) native to dry regions compared to populations native to wetter regions. While many grasses have communities of symbiotic bacteria and fungi that aid in adaptation to environmental stressors, more concerted efforts are needed to understand how endophytic microbiomes may be influenced by interactive effects of plant hosts and changing environments. Furthermore, although rhizosphere communities are easily affected by changes in the environment, they can confer stress tolerance to important prairie species such as *A. gerardii* (Sarkar *et al.* 2022). However, this phenomenon has not been studied in prairie species *Tripsacum dactyloides*. Generally, little is known about the *T. dactyloides* endophytic and epiphytic microbiomes, or how their composition changes across environments. Dissecting the rhizosphere microbiome in *T. dactyloides* can shed light upon the improvement of drought tolerance and plant productivity in grasses found in both natural and agricultural ecosystems. *T. dactyloides* is a critical species in prairie ecosystems, helps with soil stabilization, and is a valuable forage crop for animal grazing (Springer and Dewald 2004). As a perennial C4 grass, *T. dactyloides* is also projected to be able to adapt well to climate change (Norton *et al.* 2016). Additionally, *T. dactyloides* is the closest temperate relative of the globally important food crop *Zea mays* (maize), which may have important implications relating to domestication and changes in microbial associations (Kellogg and Birchler 1993).

To investigate the potential impact of historical drought adaptation on plant–microbe interactions, we quantified fungal endophytic densities in 97 *T. dactyloides* genotypes from across a steep precipitation gradient to evaluate the importance of environmental origin in structuring the symbiosis between endophytes and their host. Our common garden experimental design allowed us to disentangle the roles of the current versus historical environment. We also used amplicon sequencing to test for associations between historical precipitation and the composition of bacterial and fungal communities in roots and rhizosphere. We hypothesized that plant genotypes originally from drier sites would have a higher density of fungal endophytes in their root and leaf tissues than plants originally from wetter regions. It is well established that root endosphere microbiomes are formed as a subset of the rhizosphere soil microbiome (Lundberg *et al.* 2012; Edwards *et al.* 2015). This filtering process reflects the influence of the plant phenotype (Wagner 2021), which, in turn, is largely determined by plant genotype, which is a legacy of local adaptation in population through time. Therefore, we expected to see differences in the composition and relative abundances of microbes not only across environmental differences in the places where these plants originated from (i.e. drier vs. wetter regions) but also across sample type (i.e. soil rhizosphere vs. root endosphere).

## Methods

### Study system

**Figure 1.**
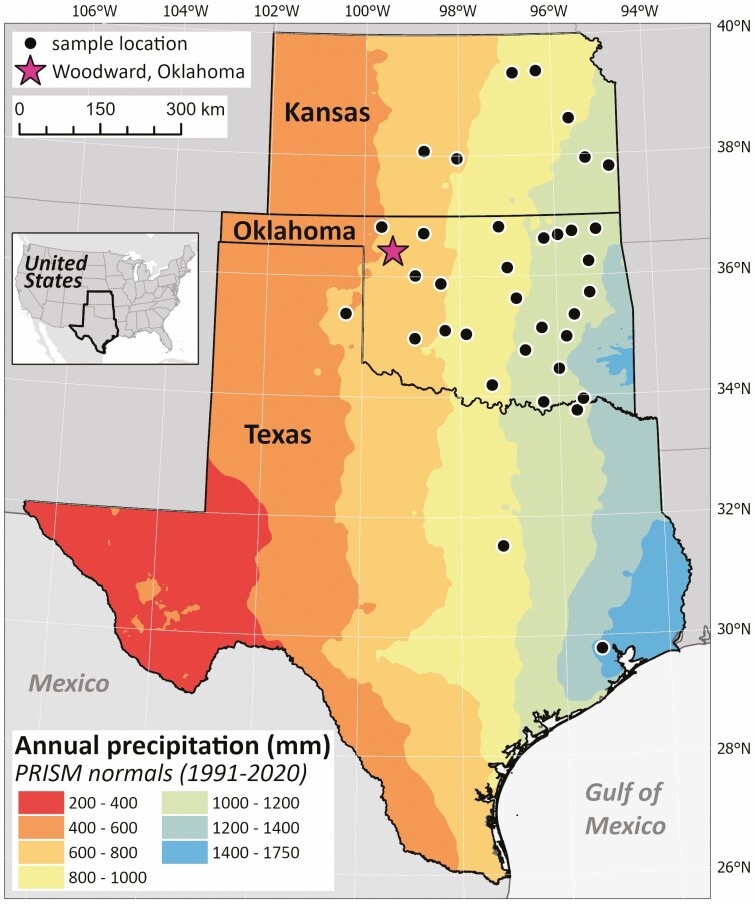
Map depicting the original collection sites of the *T. dactyloide*s genotypes. The collection sites span the east-west precipitation gradient, where MAP (mean annual precipitation) is negatively correlated with longitude (Fig. 2). The site of the common garden in Woodward, Oklahoma, is denoted by a magenta star.

**Figure 2.**
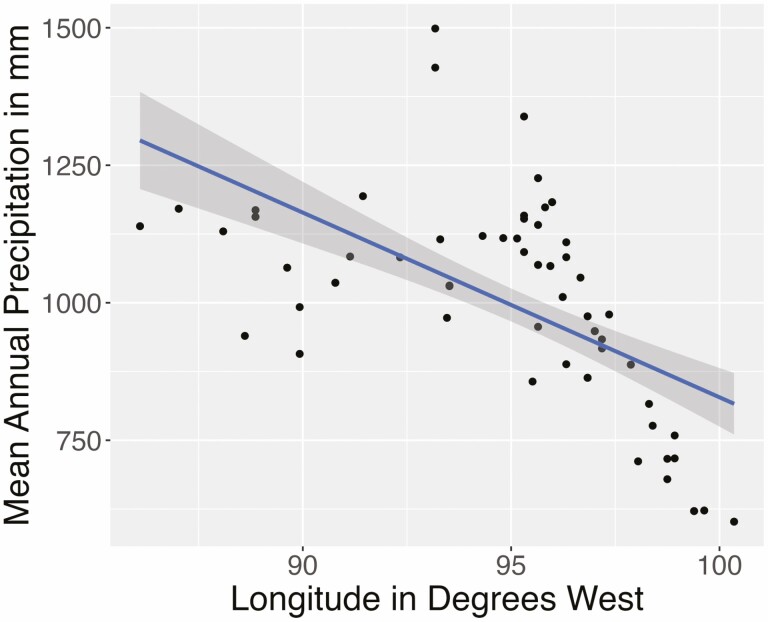
Across the area where our genotypes originated, precipitation is negatively correlated with longitude (*P* = 9.86e−11, *r* = 0.6). Each point is the original provenance of one genotype used in this study.

*Tripsacum dactyloides* (Eastern gamagrass) is a perennial C4 grass native to much of the tallgrass prairie and is known for its drought tolerance, as well as its ability to withstand periodic flooding (Springer and Dewald 2004). *T. dactyloides* reproduces vegetatively from fibrous rhizomes that are readily separable and allow for clonal propagation (Springer and Dewald 2004). This clonal reproductive process makes *T. dactyloides* an ideal candidate for studying genetic and environmental interactions, as a single genotype can be easily replicated in one or more environments. *T. dactyloides* is native to most of eastern North America and is common across Kansas, Oklahoma and Texas. These states coincide with an east–west precipitation gradient, in which western locations of the Great Plains consistently receive less precipitation than eastern areas of the Great Plains. The mean annual precipitation (MAP) in the eastern region of interest is about 381–508 mm a year, while the westward precipitation averages about 1270–1524 mm a year (Fig. 1). We chose *T. dactyloides* as a study system because it naturally occurs across this precipitation gradient and allows us to examine the effect of the environmental origin on each plant and its associated microbiome (Fig. 2).

### Field collections

To sample a breadth of genetic diversity within *T. dactyloides*, we used a collection of 97 genotypes from populations across several states including Texas, Oklahoma and Kansas (Fig. 1). These genotypes had been growing at the Southern Plains Range Research Station germplasm repository in Woodward, OK for over 20 years. Each genotype had been tagged and kept track of over many years. Weeding was also done to prevent seedling establishment in the common garden. Because the plants had been growing in a common garden for this time span, any differences between them would most likely be due to genotype since the environment was held constant. We acknowledge, however, that maternal effects influenced by the original environments of these genotypes may also have contributed to phenotypic variation.

In July 2020, we collected rhizosphere, root and leaf samples from one individual for each of the 97 genotypes in the Woodward, OK common garden (Fig. 1). The leaf samples were used for endophyte quantification via microscopy only; two separate roots were collected for amplicon sequencing and microscopy. Each leaf sample, which consisted of 12–15 cm of tissue measured from the base of the leaf, was promptly stored in 95 % ethanol until it could be further processed at the University of Kansas. Also for microscopy, we identified a <15 cm secondary lateral root and used stainless steel scissors to cut off a 10 cm segment (measured from the root tip) that also was stored in 95 % ethanol. The root collected for amplicon sequencing, was a smaller, intact tertiary lateral root (~2.5 cm long), which we cut off from the plant with scissors and placed in a 1.5 mL Eppendorf tube. Additionally, we collected the rhizosphere soil immediately surrounding this smaller root into separate 1.5 mL tubes. We used 90 % ethanol to sterilize the scissors in between samples. The small root samples and rhizosphere samples were stored on ice until they could be transferred to −20 °C for storage until DNA extraction (below).

### Endophyte quantification

The protocol for fungal endophyte quantification was adapted from McGonigle *et al.* (1990). Briefly, larger intact roots (~10 cm) that had been stored in 95 % ethanol were washed in deionized water, cut into approximately 2.5 cm sections and left in 10 % KOH solution for 5 days to clear hardy tissues. Roots were then acidified in 1 % hydrochloric acid overnight and dyed in aniline blue stain solution for 2 days. The dyed roots were left on a shaker at 30 RPM in de-stain solution consisting of 14 parts 88 % lactic acid, 1 part 100 % glycerol and 1 part water for 2–3 days. Leaves that had been stored in 95 % ethanol were washed in deionized water and cut into approximately 1 × 1 cm^2^ sections. They were then left in 1 part 100 % acetic acid and 3 parts 100 % ethanol clearing solution overnight. This clearing solution was removed the following day, and replaced with a second clearing solution composed of 1 part 100 % acetic acid, 5 parts 100 % ethanol and 1 part 100 % glycerol. The following day, the second clearing solution was replaced with aniline blue stain solution for 2 days. The dyed leaves were left on a shaker at 30 RPM in a 60 % glycerol-destaining solution for 2–3 days. Finished roots and leaves were then mounted onto slides and sealed with de-stain and a slide cover.

**Figure 3.**
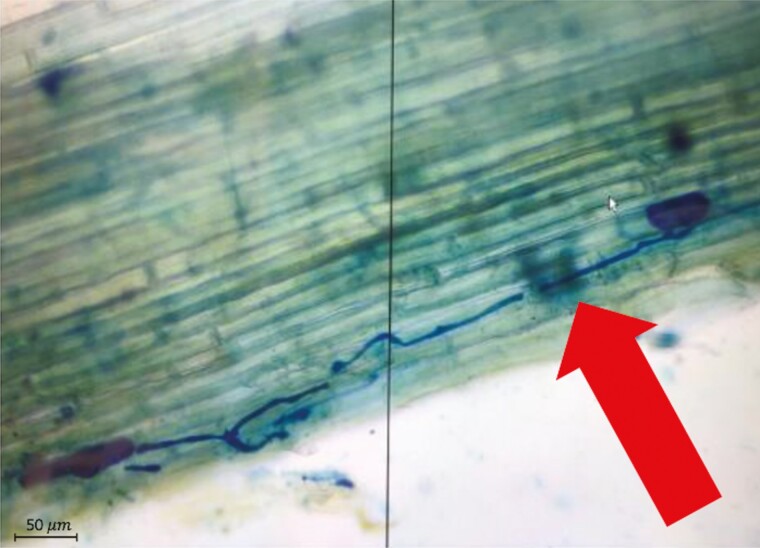
A magnified view of an endophyte-positive *T. dactyloides* leaf. The fungal hyphae have been dyed and are shown in dark blue.

One hundred fields of vision from each sample were viewed under the microscope (Accu-Scope 3000-LED, Accu-Scope INC., Commack, NY 11725) at 20× magnification. Roots and leaves were scored using the attached Manual-AU-600-Excelis HD Camera. If any fungal hyphae intersected the microscope’s crosshairs at any point in the field of vision, the field was considered endophyte positive (Fig. 3). While many of the hyphal structures were aseptate, which leads us to believe that this collection of *T. dactyloides* was heavily infected by AMF, we wanted to quantify the overall density of colonization and therefore counted all types of fungal hyphae, septate or aseptate. If the fungal structures did not intersect the microscope’s crosshairs at any point in the field of vision, it was considered endophyte negative. For each sample, we then calculated the percent fields of vision that were endophyte positive. We used this quantification method to score both leaves and roots.

### DNA extraction

To extract DNA from the root endosphere, the intact tertiary lateral roots (~2.5 cm) that had been stored at −20 °C were vortexed in lysis buffer (100 mM NaCl, 10 mM EDTA, 10 mM Tris, pH 8.0) to dislodge rhizosphere soil. Roots were washed in deionized water, freeze-dried and ground into a fine powder with steel balls in cluster tubes. Ground root tissue was deposited into wells of 2.0 mL 96-well deep plate loaded with sterile 1.0 mm garnet beads (BioSpec., Bartlesville, OK 74003). Eight hundred microlitres of lysis buffer and 250 mg of soil were placed in a 2.0 mL 96-well deep plate along with sterile garnet rocks (1.0 mm). To each well of every plate, 10 µL of 20 % SDS was added for both soil rhizosphere and root endosphere samples, and plates were homogenized at 20 Hz for 20 min. Samples were incubated at 55 °C for 90 min before being centrifuged (4500 × *g* for 6 min). Four hundred microlitres of the supernatant was transferred to a sterile 1.1 mL round bottom 96-well plate containing 130 µL of 5 M potassium acetate in each well. These plates were vortexed and incubated for 30 min at −20 °C. After incubation, the plates were thawed and centrifuged (4500 × *g* for 6 min). Four hundred microlitres of the supernatant were transferred to each well of a sterile 1.1 mL round bottom 96-well plate with 600 µL of solid phase reversible immobilization bead solution (Rohland and Reich 2012) and plates were vortexed. After allowing the DNA to bind to the beads for 10 min, plates were centrifuged again (4500 × g for 6 min) and were placed on a magnetic rack for 5 min. The resulting supernatant was removed from plates and samples were washed twice with 900 µL of ethanol. After the final wash, ethanol was removed and the plates were allowed to air dry before 75 µL of 1× Tris-EDTA buffer (pH 7.5), preheated to 37 °C, was added to each well to elute DNA from the beads. The plate was vortexed and placed on a magnet rack to allow the beads to bind, and then the supernatant was transferred to a sterile 0.45 mL 96-well place and stored at −20 °C.

### PCR and amplicon sequencing

To prepare libraries for 16S-v4 rRNA gene sequencing, 0.4 µL of forward and 0.4 µL of reverse primers 515f and 806r, respectively, were used (Apprill *et al.* 2015; Parada *et al.* 2016). For ITS1 sequencing, 0.4 µL of forward primer ITS1f and 0.4 µL of reverse primer ITS2 were used (Smith and Peay 2014). Both PCR protocols for 16S and ITS amplification also utilized 5 µL per reaction of DreamTaq Hot Start PCR Master Mix (Thermoscientific). PCRs for the amplification of 16S genes also included 0.15 µL of 100 µM of peptide nucleic acids (PNA) per reaction to suppress the amplification of the 16S sequences of chloroplasts and mitochondria (Lundberg *et al.* 2013). We used 2.5 µL of template DNA for each reaction, for a total volume of 10 µL per reaction. The 16S PCR included an initial 2-min denaturing cycle, followed by 27 cycles of denaturing for 20 s, PNA annealing for 5 s, primer annealing at 20 s and an extension for 50 s, at 95 °C, 78 °C, 52 °C and 72 °C, respectively. This was followed by a final 10-min extension at 72 °C. The ITS1 PCR included a 2-min denaturing cycle at 95 °C, followed by 27 cycles of denaturing for 20 s, primer annealing for 20 s and an extension for 50 s at 95 °C, 50 °C and 72 °C, respectively. This was followed by a 10-min extension at 72 °C. Both the 16S and ITS plates then underwent a second PCR to attach Illumina adapters with indexes. For this PCR, we used 0.8 µL of 10 µM combined forward and reverse barcoded primers with P5 and P7 Illumina adaptors. Each sample had a unique combination of eight base pairs and a binding site for annealing to amplicon sequences. This indexing PCR also utilized 5 µL per reaction of DreamTaq Hot Start PCR Master Mix, 0.15 µL of 100 µM of PNA and 1 µL of template DNA for a total volume of 10 µL. This PCR step began with a 2-min denaturing cycle, followed by 8 cycles of denaturing for 20 s, PNA annealing for 5 s, primer annealing at 20 s and an extension for 50 s, at 95 °C, 78 °C, 52 °C and 72 °C, respectively. This was followed by a 10-min extension at 72 °C. 10 µL of each reaction was pooled, keeping bacteria and fungi separate. Each pool was normalized using the ‘Just-a-Plate’ kit (Charm Biotech). DNA was quantified using QuantiFluor reagents and a Promega Quantus fluorometer. Pools were combined in equal molarity and sequenced on the Illumina platform Novaseq 6000 at 250 bp PE.

### Sequence processing and quality filtering

Cutadapt was used to sort and trim 5’ gene primers from forward and reverse reads in parallel for both bacteria and fungi (Martin 2011). We used Dada2 v.1.20 to filter out reads with 2 or more errors or ambiguous bases, and then truncated both forward and reverse 16S reads at 210 base pairs (Callahan *et al.* 2016). We used 1 × 10^8^ bases to infer error rates for both fungi and bacteria, then denoised the reads to classify amplicon sequence variants (ASVs). We then used the RDP training set (v16) to assign taxonomy to bacterial ASVs and the UNITE database to assign taxonomy to fungal ASVs (Cole *et al.* 2014; Nilsson *et al.* 2019).

We excluded ASVs that were identified as plant sequences, as well as ASVs whose kingdom could not be classified. Our filtering processes reduced the number of bacterial ASVs from 13911 to 2297 and the number of fungal ASVs from 603 to 67. We discarded any ASVs that were not present in at least 25 reads in at least 3 samples. The ASVs that were thrown out at this stage were rare or assigned due to a sequencing error, therefore, we retained 86.5 % and 87.7 % of bacterial and fungal reads, respectively. We removed samples with fewer than 400 and 150 usable reads for bacteria and fungi, respectively. We then used R package ALDEx2 to apply a centred log ratio (CLR) transformation to the remaining observations in each sample, to stabilize the variance and account for the compositional nature of our data (Fernandes *et al.* 2014; Gloor *et al.* 2017). After quality control, we were able to analyse bacterial and fungal microbiome composition for 48 and 29 genotypes, respectively.

### Data analysis

We used R (v. 4.2.2) for all our data analyses with the packages phyloseq, vegan, genefilter, ALDEx2 and tidyverse (Fernandes *et al.* 2013, 2014; Love *et al.* 2014; McMurdie and Holmes 2014; Gloor *et al.* 2016; R. Gentleman 2017; Wickham *et al.* 2019). For our endophyte quantification analysis, we used the following linear models: *root fungal density or leaf fungal density ~ precipitation*. For our microbiome analyses, we calculated the relative abundances using the raw microbiome counts. We used the following negative binomial regression to compare the relative abundances of taxa between sample types for both bacterial and fungal taxa: *glm.nb(abundance ~ sample type (rhizosphere soil or root endosphere)* + *sequencing depth(to control for noise attributable to variation in sequencing depth)).* We only modelled the set of taxa that were present in both groups. We used transformed data to calculate alpha diversity metrics, Inverse Simpson and Shannon indices, from the untransformed data using VEGAN (Oksanen *et al.* 2022). These metrics were modelled using the following explanatory variables: *Inverse Simpson or Shannon ~ sample type (rhizosphere soil or root endosphere)* + *sequencing depth* and *Inverse Simpson or Shannon* *~* *historic Mean annual precipitation (MAP)* + *sequencing depth* and assessed the model using ANCOVA. *P*-values were adjusted to account for multiple comparisons (Benjamini and Hochberg 1995). We also ran a canonical analysis of principal components (CAP) ordination using Aitchison distance of both bacterial and fungal communities, using sample type (i.e. root endosphere vs. rhizosphere soil) to constrain the ordination, and controlled for sequencing depth after applying a CLR transformation to the sequencing counts. We ran a second CAP ordination using Aitchison distance on CLR–transformed bacterial and fungal taxa, this time using precipitation at the place of origin where the sample was collected from to constrain the ordination, and once again controlled for sequencing depth. We then used a linear model to determine whether the CLR-transformed counts of both bacterial and fungal taxa varied across the precipitation gradient, while once again controlling for variations in sequencing depth: *lm(Abundance* *~* *historic MAP* + *sequencing depth).*

## Results

### Endophyte quantification

**Figure 4.**
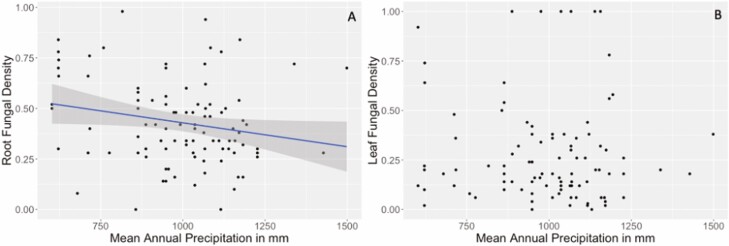
(A) Root endophyte density as a function of MAP (millimetres) at each genotype’s site of origin (*P* = 0.043). Leaf endophyte density as a function of MAP (millimetres) at each genotype’s site of origin (*P* = 0.62). We define density as the percentage of fields of vision that were endophyte positive (see ‘Methods’ section). Each dot represents an individual sample, and the lines are the regression lines of best fit. The grey shading represents the 95 % confidence interval.

Roots and leaves of 97 genotypes were cleared and stained to quantify internal fungal structures via microscopy. Genotypes originating from habitats with lower MAPs had higher densities of root endophytes (Fig. 4A, *F* = 4.202, df = 1, 94, *P* = 0.043).

In contrast, leaf tissue endophyte densities were not correlated with the precipitation (Fig. 4B, *F* = 0.2509, df = 1, 94, *P* = 0.62) of the genotype’s collection site.

### Amplicon sequencing

**Figure 5.**
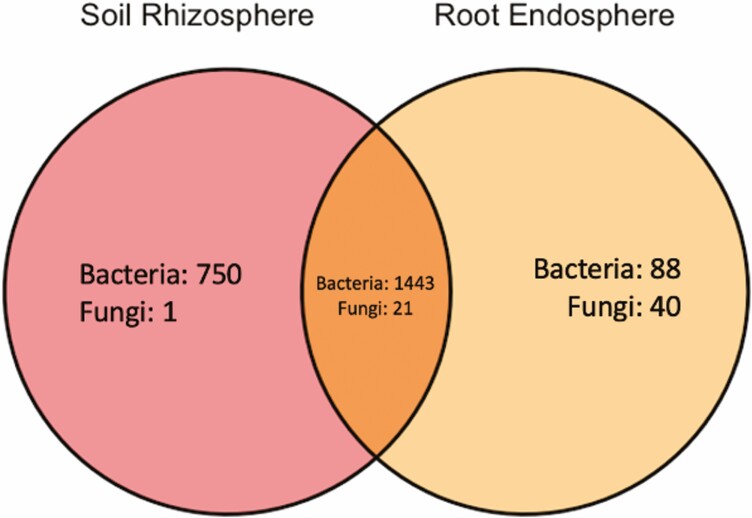
Venn diagram portraying the number of taxa that were unique to and shared between soil rhizosphere and root endosphere.

We used amplicon sequencing to characterize the bacterial and fungal communities of *T. dactyloides*. Out of 2297 total bacterial taxa sequenced from our samples, 750 were unique to the soil rhizophere, 88 were unique to the root endosphere and 1443 were common between the two sample types (Fig. 5). Out of 62 fungal taxa sequenced, 1 was unique to the soil rhizosphere, 40 were unique to the root endosphere and 21 were common between both sample types.

**Figure 6.**
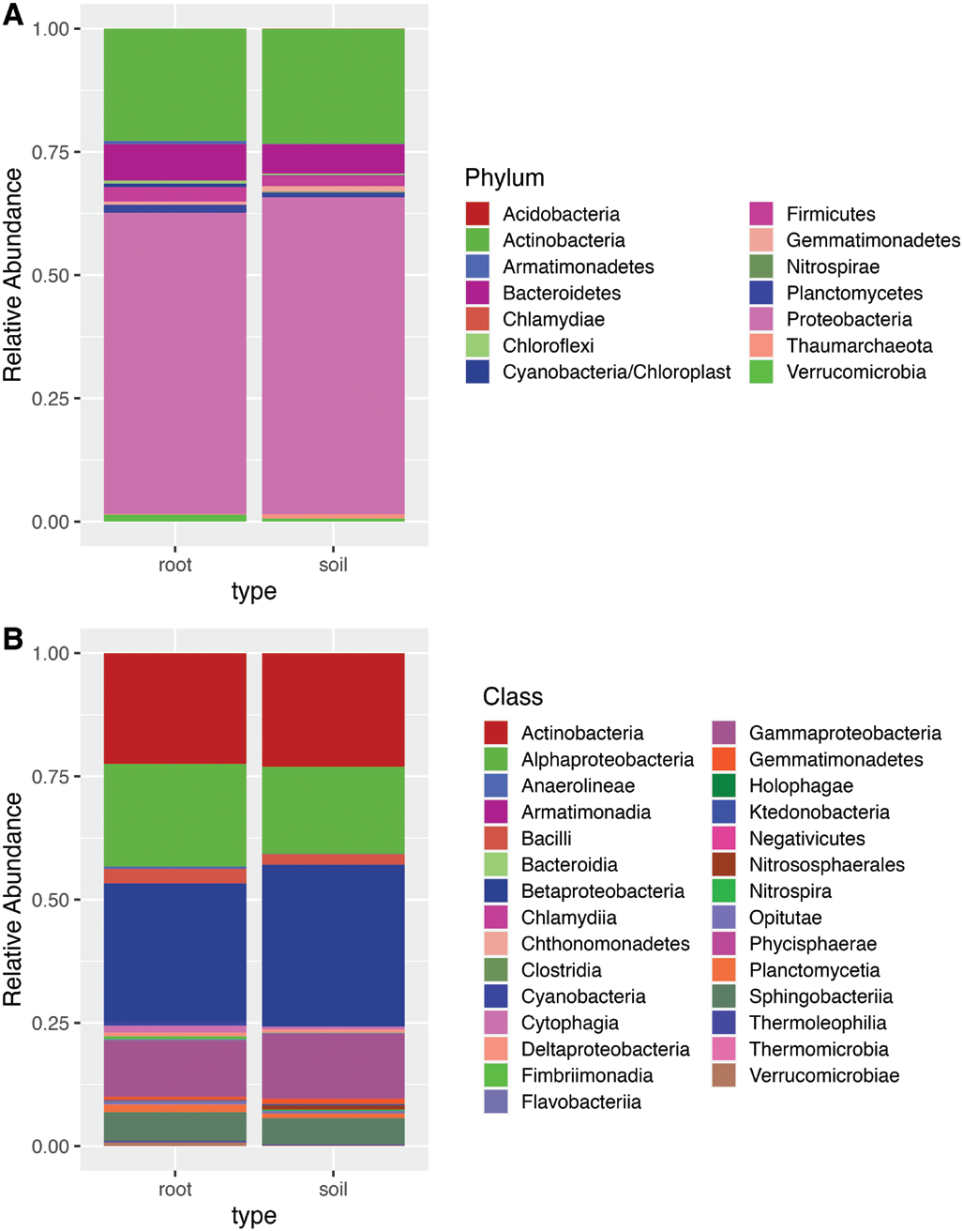
Relative abundances of bacterial phyla (A) and classes (B) found in 48 samples of *T. dactyloides*. We have separated the root endosphere sequences (first panels) and the soil rhizosphere samples (second panels).

Firmicutes and Verrucomicrobia were more abundant in the root endosphere than rhizosphere (2.9 % to 2.1 % respectively, *P* < 0.001) and (1.4 % to 0.63 %, respectively, *P* = 0.02, Fig. 6A). Proteobacteria were more abundant in rhizosphere than root endosphere (64.2 % vs. 61.1 %, *P* < 0.001). The most abundant class of bacteria recovered were Betaproteobacteria, followed by Actinobacteria and Alphaproteobacteria (Fig. 6B). Betaproteobacteria were relatively more abundant in rhizosphere than root endosphere (*P* < 0.001).

**Figure 7.**
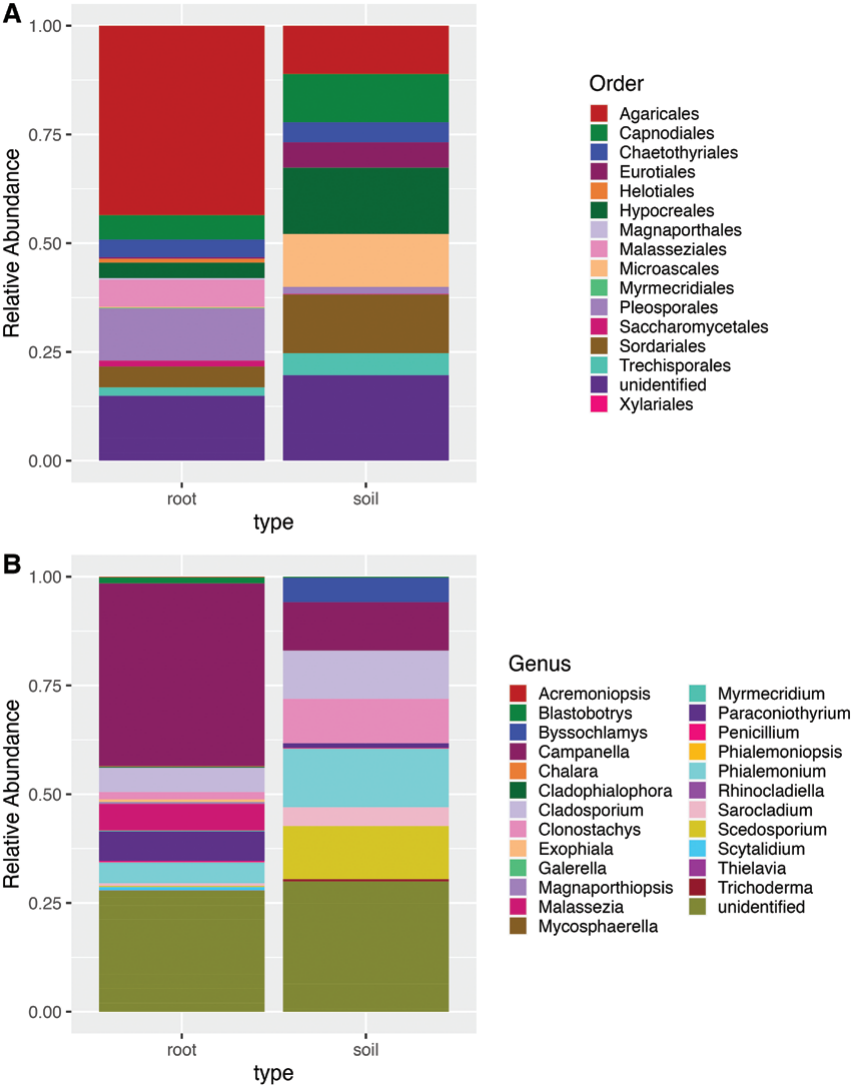
Relative abundances of fungal orders (panel A) and genera (panel B) in 23 samples of *T. dactyloides*. We have separated the root endosphere sequences and the rhizosphere samples.

Fungi were not sequenced as successfully as bacteria and many unusable or non-reproducible reads were filtered out. On the order level, Hypocreales were more abundant in rhizosphere than root endosphere (15.2 % and 3.56 %, respectively, *P* < 0.001, Fig. 7A). Pleosporales were more abundant in our root endosphere samples than in our rhizosphere samples. (1.53 % and 11.9 % respectively, *P* < 0.001).

**Figure 8.**
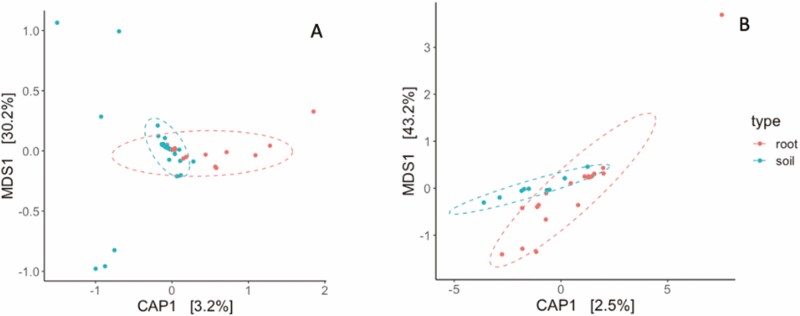
CAP ordination for CLR-transformed bacterial (A) and fungal (B) samples, constrained by sample type (i.e. root endosphere vs. rhizosphere). Neither bacterial nor fungal community composition differed between root and rhizosphere (*P* > 0.05).

The diversity of bacterial ASVs in the root endosphere and rhizosphere samples did not significantly differ when measured using the Inverse Simpson index or the Shannon Index (Supporting Information—Table S1). Similarly, fungal diversity did not significantly vary between endosphere and rhizosphere samples, as determined by both the Inverse Simpson and Shannon indices (Supporting Information—Table S2). Neither bacterial nor fungal diversity shifted along the precipitation gradient (Supporting Information—Tables S1 and S2, respectively). A CAP ordination constrained by sample type (i.e. root endosphere vs. rhizosphere) suggested that there was no significant difference in bacterial community composition between the root endosphere and rhizosphere samples (Fig. 8A, *P* > 0.05). Similarly, we found no evidence that fungal community composition differed between the root endosphere and soil rhizosphere samples (Fig. 8B, *P* > 0.05).

### Unlike endophyte density, microbiome composition did not co-vary with genotypes’ provenances along the precipitation gradient

**Figure 9.**
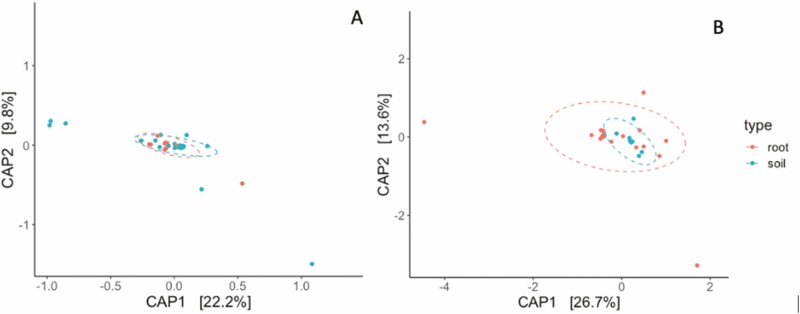
CAP ordination for CLR-transformed bacterial (A) and fungal (B) samples, constrained by precipitation at the genotype’s original location. Root and rhizosphere bacterial or fungal communities did not co-vary with genotypes’ provenances (*P* > 0.05) across the precipitation gradient.

A CAP ordination constrained by the MAP of the location where each sample was originally collected was also conducted on both CLR-transformed bacterial and fungal taxa. There was no significant difference in bacteria or fungal communities along the precipitation gradient (Fig. 9, *P* > 0.05). Similarly, our linear models did not detect any relationship between the relative abundances of individual bacteria or fungi and the east–west precipitation gradient (*P* > 0.05).

## Discussion

Our study investigated both absolute and relative abundances of plant-associated microbes, and how these abundances change as functions of the host genotype’s historical location across a regional precipitation gradient. Notably, we found that historic precipitation affects absolute density (i.e. fungal endophyte densities in roots) but not the composition of either bacterial or fungal microbiomes. This finding is consistent with recent reports that genetic variation in *T. dactyloides* phenotypic traits also correlates with mean annual precipitation at each genotype’s historical location (Kiniry *et al.* 2023). Previous studies have demonstrated that environment is one of the biggest predictors of microbiome composition (Wagner *et al.* 2016; Hamonts *et al.* 2017), suggesting that our plants’ shared common garden environment masked more subtle patterns of microbiome variation. Another factor that could have affected the composition of the microbiome is seasonality, as it has been shown that microbiomes shift across seasons (Thongsandee *et al.* 2012; Deyett and Rolshausen 2019; Giampetruzzi *et al.* 2020). As we only sampled the microbiome at one time-point (mid-summer 2020), we cannot assess how the plants modified their associated microbial communities across time. AMF are difficult to identify using sequencing methods (Delavaux *et al.* 2021), and we did not see any present in our sequencing data. This may be why endophyte density, which likely included many AMF, varied across the precipitation gradient, whereas we saw no such correlation in our sequencing data.

The biggest predictor of microbiome relative abundances and composition in any of our analyses was sequencing depth. Stochastic variation in sequencing depth decreased the signal-to-noise ratio, as is typical for amplicon-sequencing studies. Nevertheless, our analyses reveal an overall theme that the host genotype’s origin along the precipitation gradient predicts the absolute abundance of symbionts in the root, but not the relative abundance of particular organisms nor the overall community composition.

We observed higher densities of fungal endophytes in the roots of genotypes from western, drier origins, compared to genotypes from eastern, wetter regions, but leaf samples did not show a similar pattern. As a perennial plant, the belowground structures (i.e. roots) of *T. dactyloides* remain alive year-round, with a period of dormancy during the winter season. In contrast, aboveground structures (i.e. leaves) senesce during winter and are newly formed each year. Due to this, root microbial communities in perennial plants have been found to be relatively stable year over year (Chialva *et al.* 2021). In contrast, the annually produced leaves of perennial plants must start with a fresh microbiome each year, making them more susceptible to stochastic influences and resulting in less stability in their microbial composition (Wagner *et al.* 2016). This observation may explain the greater influence of the host plant on root endophyte composition over time, as compared to the less stable leaf endophyte communities that die annually and have less opportunity to be affected by their host plant.

While the ability of fungal root endophytes to enhance the drought tolerance of their hosts has been well studied (Begum *et al.* 2019), the potential role of fungal leaf endophytes in enhancing host drought tolerance remains largely unexplored, except for one well-known genus. The fungal genus *Epichloë* consists of vertically transmitted endophytes that are associated with less water use and tolerance to stresses, such as heavy metal and herbivory, in host plants (Verma *et al.* 2022). However, *Epichloë* is primarily known to infect C3 grasses (but see Marks and Clay 1990). Whether or not fungal leaf endophytes could potentially play a role in the drought tolerance of *T. dactyloides* beyond the scope of our results is currently unknown.

Because we found no evidence for a relationship between microbiome composition and precipitation at the host genotype’s site of origin, we focussed our analyses on comparing the composition and abundance of individual taxa between the soil rhizosphere and the root endosphere communities of our samples. It is well-documented that plant phenotype (such as excreted chemical exudates) influences which microbes enter the root endosphere, and accumulate in the rhizosphere soil (Bulgarelli *et al.* 2013; Wagner 2021). Therefore, the differences in the relative abundances of certain taxa between soil rhizosphere and root endosphere may reflect the extent to which the plants sampled were curating their associated microbiomes. Notably, many of these taxa have been reported to have dramatic impacts on the health or productivity of their host plants.

For example, the major classes of bacteria found in our samples of *T. dactyloides* serve a multitude of functions in host plants. One of the most abundant taxa, Actinobacteria, has been found to protect the host plant from pathogens and aid in converting nitrogen and phosphorous to more usable forms (Bhatti *et al.* 2017). This phylum was found in almost equal relative abundance in rhizosphere and root endosphere in our samples of *T. dactyloides*, at 23.2 % and 22.7 %, respectively. Interestingly, this phylum was enriched in response to drought treatment in rice (Santos-Medellín *et al.* 2021). Some strains in the class Bacilli also aid in nutrient conversion and protection from disease (Miljaković *et al.* 2020). Alphaproteobacteria includes symbiotic strains with many relevant functions, such as plant growth promotion, and also includes some non-symbiotic strains (Pini *et al.* 2011). This class was more abundant in *T. dactyloides* roots than in rhizosphere (*P* < 0.001), perhaps due to its role as a plant growth promoter, making it more useful inside the plant roots. This finding was consistent with previous research demonstrating an enrichment of Alphaproteobacteria in the endosphere relative to the rhizosphere (Beckers *et al.* 2017). The presence of some Gammaproteobacteria in the microbiome has been associated with crop plant health (Köberl *et al.* 2017). We found a higher abundance of this taxon in the rhizosphere compared to root endosphere in our samples (*P* < 0.001), as did Beckers *et al.* (2017) in poplar trees. Betaproteobacteria, the most abundant class present in our samples, has been shown to contribute to nitrogen, sulfur and carbon cycling, protection against pathogens, as well as the production of stress-reducing enzymes (İnceoğlu *et al.* 2010). This taxon was also more abundant in the *T. dactyloides* rhizosphere, where it may play a greater role in nutrient cycling than it would inside the root endosphere or even in bulk soil (Li *et al.* 2014).

Similarly, the most abundant fungal genera identified also have a range of functions, ranging from symbiotic to saprotrophic. The genus *Blastobotrys* contains a non-pathogenic yeast that is both heat- and salt-tolerant (Malak *et al.* 2016). Species in the *Byssochlamys* genus have been found to protect perennial grass *Lolium rigidum* from pathogens (Rodrigo *et al.* 2017), while *Clonostachys* is an endophytic mycoparasite that can establish itself close to locations along the plant’s surface where pathogens may be able infect (Funck Jensen *et al.* 2021). We found this taxon in higher abundance in the rhizosphere (*P* < 0.001), where it may be more useful in proactively obstructing pathogens from entering the root endosphere (Fig. 6B). *Paraconiothyrium* spp. causes cane blight in many crop species (Guarnaccia *et al.* 2022), whereas *Cladosporium* spp. and *Phialemonium* spp. can both protect against pathogens and promote plant growth in various crop species (Jiang *et al.* 2021; Rivera-Vega *et al.* 2022). We found both of these taxa in higher abundance in the rhizosphere (*P* < 0.001 each), but they were nonetheless also present in the root endosphere (Fig. 6B). However, some species of *Cladosporium* are parasitic or saprotrophic (Bensch *et al.* 2012). Species of the genus *Sarocladium* produce sheath rot disease in rice (Liu 2017), but produce antibiotics that protects maize kernels from pathogens (Wicklow *et al.* 2005). *Scytalidium* spp. was found to influence root architecture in a way that protected plants from heavy metal contamination in the soil, and also promotes plant growth under the same conditions (Hou *et al.* 2020). While we have no evidence that our *T. dactyloides* plants were exposed to heavy metal contamination, *Scytalidium* sp. was found in the root endosphere but not the rhizosphere, perhaps congruent with the findings on root architecture influences and plant growth promotion in the aforementioned study.

Because *Tripsacum* is the sister genus to *Zea* spp., our results may be informative about processes of microbiome assembly in the globally important grain crop, maize (*Zea mays mays*)--especially in the U.S. Corn Belt, which is within the native range of *T. dactyloides*. Therefore, we compared the microbiome composition of *T. dactyloides* to those of maize and similar and related plants, including switchgrass (*Panicum virgatum*) and teosinte (*Zea mays parviglumis*), the progenitor to maize. Similar to our data from *T. dactyloides*, the microbiome of *P. virgatum* is dominated by the bacteria phyla Firmicutes, Proteobacteria and Actinobacteria (Hestrin *et al.* 2021). Specifically, within the phylum Proteobacteria, both Gammaproteobacteria and Alphaproteobacteria comprise much of the bacterial microbiome of both *T. dactyloides* and *P. virgatum* (Hestrin *et al.* 2021). Fungal orders that have been found in the microbiome of both species include Capnodiales, Eurotiales, Helotiales, Hypocreales, Pieosporales, Sordariales and Xylariales (Hestrin *et al.* 2021). Similar to *T. dactyloides*, *P. virgatum* is also regarded as a drought-tolerant species (Barney *et al.* 2009), although more work is needed to determine whether this shared trait is related to the similarity in their microbial associations. Teosinte and maize are both closely related to *T. dactyloides* (Kellogg and Birchler 1993). The maize microbiome differs from that of teosinte due to domestication changing root architecture and chemical exudates, leading to changes in the microbe–host association (Pérez-Jaramillo *et al.* 2016). Alphaproteobacteria, Gemmatimonadetes and Actinobacteria make up a much of the bacterial microbiome of both teosinte and *T. dactyloides*, while Betaproteobacteria is abundant in both maize and *T. dactyloides* (Brisson *et al.* 2019). Betaproteobacteria has been shown to be more abundant in maize soil rhizosphere specifically, compared to bulk soil (Li *et al.* 2014). As for fungi, *Cladosporium* appears to be the only genus found in these samples of *T. dactyloides*, and in both teosinte and maize (Brisson *et al.* 2019). Teosinte has long been studied for its potential to improve inbred lines of maize, specifically by increasing its tolerance to drought and other stresses (Reeves 2010). The overlap in microbiome membership between teosinte and *T. dactyloides* raises the possibility of microbial interactions as a mechanism of drought tolerance.

Findings from this research could be applied to conservation work in the tallgrass prairie ecosystem to help ensure healthy function. The North American tallgrass prairie lands are increasingly threatened by more frequent and severe droughts as global climate change increases average temperatures. Decades of fire suppression, ploughing under native grasses and the replacement of diverse, native, plants with monocultures have significantly altered the indigenous soil and plant microbial communities. A 5-year-long study showed that fungal and bacterial treatments to a desertified shrubland helped improve soil nutrient content and aggregation, as well as helped establish new plant growth and jump-start the process of ecological succession (Requena *et al.* 2001). Similarly, a different study demonstrated that AMF inocula from nearby native prairie remnants helped increase both plant diversity and richness when transplanted in a plot undergoing restoration (Koziol and Bever 2017). Additionally, a greater understanding of the symbiosis between plants and their microbial communities is essential for developing more tolerant crops as agricultural systems are impacted by global shifts in climate patterns (Trivedi *et al.* 2017). An inoculation of both AMF and plant growth-promoting bacteria in maize helped mitigate environmental stressors, as well as improved crop production (Moreira *et al.* 2020).

Altogether, the evidence from other, better-studied plant systems supports further research into the functions of the *T. dactyloides* microbiome. Our results show that *T. dactyloides* appears to associate with microbes that may assist with several functions that could be threatened by a changing climate, such as nutrient cycling, protection against pathogens and stress reduction and tolerance (İnceoğlu *et al.* 2010; Malak *et al.* 2016). One major limitation of amplicon-sequencing data is the difficulty of inferring microbiome function from taxonomic composition, which limits the direct usefulness of this particular dataset. To remedy this, however, we have initiated a follow-up study that uses plant phenotype to quantify microbiome function directly, both in general and in a genotype-dependent context. Further study into the taxa discussed in this paper could lead to discoveries in beneficial microbe inoculation that have been previously used in both agriculture and restoration (Koziol and Bever 2017; Santos *et al.* 2019). Finally, our observation that genetic variation in plant–microbe interactions corresponds geographically to historical precipitation raises the possibility that belowground microbiomes have contributed to local adaptation in *T. dactyloides* and thus supports the importance of considering the genetic origins of plants used in conservation and restoration work—particularly in regions that are at risk of drought.

## Supporting Information

The following additional information is available in the online version of this article –

**Table S1.** Bacterial alpha diversity metrics. Neither Inverse Simpson diversity nor Shannon diversity significantly vary between sample type (root endosphere or soil rhizosphere) or across mean annual precipitation of the location of origin. *P*-values were adjusted to account for multiple comparisons.**Table S2.** Fungal alpha diversity metrics. Neither Inverse Simpson diversity nor Shannon diversity significantly vary between sample type (root endosphere or soil rhizosphere) or across mean annual precipitation of the location of origin. *P*-values were adjusted to account for multiple comparisons.

Tables 1 and 2 are both found in the Supporting Information Document.

plad072_suppl_Supplementary_Tables_S1-S2

## Data Availability

All code and microscopy data are available on Zenodo (DOI: 10.5281/zenodo.8400116). Sequence data are available on NCBI SRA (BioProject number PRJNA1026895). The accession information is available in the SRA Biosample data and in the same Zenodo dataset.
