# Supplementary material for: Interactions with fungi vary among Tripsacum dactyloides genotypes from across a precipitation gradient
Source: AoB Plants. 2023 Nov 2;15(6):plad072. doi: 10.1093/aobpla/plad072 (PMC10667659; doi:10.1093/aobpla/plad072)
Supplement: plad072_suppl_Supplementary_Tables_S1-S2 [file plad072_suppl_supplementary_tables_s1-s2.docx]

Supporting Information for Interactions with fungi vary among *Tripsacum dactyloides* genotypes from across a precipitation gradient

| **Response** | **Term** | **Numerator Degrees of Freedom** | **Denominator Degrees of Freedom** | **F** | **P-adjusted** |
| --- | --- | --- | --- | --- | --- |
| **InvSimpson** | **type(rhizosphere**  **vs. endosphere)** | **1** | **45** | **0.70** | **0.41** |
| **InvSimpson** | **precip(mean annual**  **precipitation of location**  **of origin** | **1** | **45** | **0.44** | **0.97** |
| **InvSimpson** | **Sequencing Depth** | **1** | **45** | **18.3** | **0.0002** |
| **Shannon** | **type** | **1** | **45** | **3.23** | **0.16** |
| **Shannon** | **precip(mean annual**  **precipitation of location**  **of origin** | **1** | **45** | **0.78** | **0.97** |
| **Shannon** | **Sequencing depth** | **1** | **45** | **40.12** | **2.45E-05** |

Table 1. Bacterial alpha diversity metrics. Neither Inverse Simpson diversity nor Shannon diversity significantly vary between sample type (root endosphere or soil rhizosphere) or across mean annual precipitation of the location of origin. P-values were adjusted to account for multiple comparisons.

| **Response** | **Term** | **Numerator Degrees of Freedom** | **Denominator Degrees of Freedom** | **F** | **P-adjusted** |
| --- | --- | --- | --- | --- | --- |
| **InvSimpson** | **Type rhizosphere**  **vs. endosphere)** | **1** | **26** | **1.66** | **0.27** |
| **InvSimpson** | **Sequencing Depth** | **1** | **26** | **0.176** | **0.68** |
| **InvSimpson** | **precip(mean annual**  **precipitation of location**  **of origin** | **1** | **26** | **0.861** | **0.61** |
| **Shannon** | **type** | **1** | **26** | **1.34** | **0.27** |
| **Shannon** | **precip(mean annual**  **precipitation of location**  **of origin** | **1** | **26** | **0.934** | **0.61** |
| **Shannon** | **Sequencing Depth** | **1** | **26** | **0.339** | **0.68** |

Table 2. Fungal alpha diversity metrics. Neither Inverse Simpson diversity nor Shannon diversity significantly vary between sample type (root endosphere or soil rhizosphere) or across mean annual precipitation of the location of origin. P-values were adjusted to account for multiple comparisons.
